# Supplementary material for: Identification of urban land use efficiency by indicator-SDG 11.3.1
Source: PLoS One. 2020 Dec 28;15(12):e0244318. doi: 10.1371/journal.pone.0244318 (PMC7769485; doi:10.1371/journal.pone.0244318)
Supplement: Attachment — Submitted filename: Responds to ReviewersV.doc [file pone.0244318.s002.doc]

Dear editor and reviewers,

First of all, I would like to thank you for your critical comments on our manuscript. According to your comments, we carefully revised our manuscript. Please see detail below and the revised version.

PLOS ONE

Journal Requirements:

When submitting your revision, we need you to address these additional requirements.

1. Please ensure that your manuscript meets PLOS ONE's style requirements, including those for file naming. The PLOS ONE style templates can be found at

https://journals.plos.org/plosone/s/file?id=wjVg/PLOSOne_formatting_sample_main_body.pdf and

https://journals.plos.org/plosone/s/file?id=ba62/PLOSOne_formatting_sample_title_authors_affiliations.pdf

**Reply:** **We have revised the manuscript according to the PLOS ONE’s style requirements from your suggested templates.**

2. Thank you for submitting the above manuscript to PLOS ONE. During our internal evaluation of the manuscript, we found significant text overlap between your submission and the following previously published works, some of which you are an author.

int-arch-photogramm-remote-sens-spatial-inf-sci.net/XLII-3-W10/1073/2020/isprs-archives-XLII-3-W10-1073-2020.pdf

We would like to make you aware that copying extracts from previous publications, especially outside the methods section, word-for-word is unacceptable. In addition, the reproduction of text from published reports has implications for the copyright that may apply to the publications.

Please revise the manuscript to rephrase the duplicated text, cite your sources, and provide details as to how the current manuscript advances on previous work. Please note that further consideration is dependent on the submission of a manuscript that addresses these concerns about the overlap in text with published work.

We will carefully review your manuscript upon resubmission, so please ensure that your revision is thorough.

**Reply:** **Thanks for pointing this out. We have checked out all of the overlap with the pervious published work and revised the sentences one by one. Please see detail in the revised manuscript with track.**

3. We suggest you thoroughly copyedit your manuscript for language usage, spelling, and grammar. If you do not know anyone who can help you do this, you may wish to consider employing a professional scientific editing service.  

Whilst you may use any professional scientific editing service of your choice, PLOS has partnered with both American Journal Experts (AJE) and Editage to provide discounted services to PLOS authors. Both organizations have experience helping authors meet PLOS guidelines and can provide language editing, translation, manuscript formatting, and figure formatting to ensure your manuscript meets our submission guidelines. To take advantage of our partnership with AJE, visit the AJE website ([http://learn.aje.com/plos/](about:blank)) for a 15% discount off AJE services. To take advantage of our partnership with Editage, visit the Editage website (www.editage.com) and enter referral code PLOSEDIT for a 15% discount off Editage services.  If the PLOS editorial team finds any language issues in text that either AJE or Editage has edited, the service provider will re-edit the text for free.

Upon resubmission, please provide the following:

- The name of the colleague or the details of the professional service that edited your manuscript
- A copy of your manuscript showing your changes by either highlighting them or using track changes (uploaded as a *supporting information* file)
- A clean copy of the edited manuscript (uploaded as the new *manuscript* file)

**Reply:** **We have asked Elsevier Language Editing Services which improved my manuscript several times before edited this revised manuscript. We submitted our manuscripts with track changes and clean respectively.**

4. We note that you have indicated that data from this study are available upon request. PLOS only allows data to be available upon request if there are legal or ethical restrictions on sharing data publicly. For more information on unacceptable data access restrictions, please see http://journals.plos.org/plosone/s/data-availability#loc-unacceptable-data-access-restrictions.

In your revised cover letter, please address the following prompts:

a) If there are ethical or legal restrictions on sharing a de-identified data set, please explain them in detail (e.g., data contain potentially sensitive information, data are owned by a third-party organization, etc.) and who has imposed them (e.g., an ethics committee). Please also provide contact information for a data access committee, ethics committee, or other institutional body to which data requests may be sent.

b) If there are no restrictions, please upload the minimal anonymized data set necessary to replicate your study findings as either Supporting Information files or to a stable, public repository and provide us with the relevant URLs, DOIs, or accession numbers. For a list of acceptable repositories, please see http://journals.plos.org/plosone/s/data-availability#loc-recommended-repositories.

We will update your Data Availability statement on your behalf to reflect the information you provide.

**Reply:** **Thanks. I have made the data available online.**

5. PLOS requires an ORCID iD for the corresponding author in Editorial Manager on papers submitted after December 6th, 2016. Please ensure that you have an ORCID iD and that it is validated in Editorial Manager. To do this, go to ‘Update my Information’ (in the upper left-hand corner of the main menu), and click on the Fetch/Validate link next to the ORCID field. This will take you to the ORCID site and allow you to create a new iD or authenticate a pre-existing iD in Editorial Manager. Please see the following video for instructions on linking an ORCID iD to your Editorial Manager account: https://www.youtube.com/watch?v=_xcclfuvtxQ

**Reply:** **I have created an ORCID which is** [**https://orcid.org/0000-0001-6581-0192**](https://orcid.org/0000-0001-6581-0192)

6. We note that Figure 1 and 3 in your submission contain map images which may be copyrighted. All PLOS content is published under the Creative Commons Attribution License (CC BY 4.0), which means that the manuscript, images, and Supporting Information files will be freely available online, and any third party is permitted to access, download, copy, distribute, and use these materials in any way, even commercially, with proper attribution. For these reasons, we cannot publish previously copyrighted maps or satellite images created using proprietary data, such as Google software (Google Maps, Street View, and Earth). For more information, see our copyright guidelines: http://journals.plos.org/plosone/s/licenses-and-copyright.

We require you to either (1) present written permission from the copyright holder to publish these figures specifically under the CC BY 4.0 license, or (2) remove the figures from your submission:

6.1.    You may seek permission from the original copyright holder of Figure 1 and 3 to publish the content specifically under the CC BY 4.0 license.

We recommend that you contact the original copyright holder with the Content Permission Form (http://journals.plos.org/plosone/s/file?id=7c09/content-permission-form.pdf) and the following text:

“I request permission for the open-access journal PLOS ONE to publish XXX under the Creative Commons Attribution License (CCAL) CC BY 4.0 (http://creativecommons.org/licenses/by/4.0/). Please be aware that this license allows unrestricted use and distribution, even commercially, by third parties. Please reply and provide explicit written permission to publish XXX under a CC BY license and complete the attached form.”

Please upload the completed Content Permission Form or other proof of granted permissions as an "Other" file with your submission.

In the figure caption of the copyrighted figure, please include the following text: “Reprinted from [ref] under a CC BY license, with permission from [name of publisher], original copyright [original copyright year].”

6.2.    If you are unable to obtain permission from the original copyright holder to publish these figures under the CC BY 4.0 license or if the copyright holder’s requirements are incompatible with the CC BY 4.0 license, please either i) remove the figure or ii) supply a replacement figure that complies with the CC BY 4.0 license. Please check copyright information on all replacement figures and update the figure caption with source information. If applicable, please specify in the figure caption text when a figure is similar but not identical to the original image and is therefore for illustrative purposes only.

The following resources for replacing copyrighted map figures may be helpful:

USGS National Map Viewer (public domain): http://viewer.nationalmap.gov/viewer/

The Gateway to Astronaut Photography of Earth (public domain): http://eol.jsc.nasa.gov/sseop/clickmap/

Maps at the CIA (public domain): https://www.cia.gov/library/publications/the-world-factbook/index.html and https://www.cia.gov/library/publications/cia-maps-publications/index.html

NASA Earth Observatory (public domain): http://earthobservatory.nasa.gov/

Landsat: http://landsat.visibleearth.nasa.gov/

USGS EROS (Earth Resources Observatory and Science (EROS) Center) (public domain): http://eros.usgs.gov/#

Natural Earth (public domain): http://www.naturalearthdata.com/

**Reply: We replaced Figure 1 by screened USGS National Map Viewer image and removed images in Figure 3 while remaining only the built-up lands.**

[Note: HTML markup is below. Please do not edit.]

Reviewers' comments:

Reviewer's Responses to Questions

Comments to the Author

1. Is the manuscript technically sound, and do the data support the conclusions?

The manuscript must describe a technically sound piece of scientific research with data that supports the conclusions. Experiments must have been conducted rigorously, with appropriate controls, replication, and sample sizes. The conclusions must be drawn appropriately based on the data presented.

Reviewer #1: Yes

Reviewer #2: Partly

2. Has the statistical analysis been performed appropriately and rigorously?

Reviewer #1: Yes

Reviewer #2: Yes

3. Have the authors made all data underlying the findings in their manuscript fully available?

The [PLOS Data policy](http://www.plosone.org/static/policies.action#sharing) requires authors to make all data underlying the findings described in their manuscript fully available without restriction, with rare exception (please refer to the Data Availability Statement in the manuscript PDF file). The data should be provided as part of the manuscript or its supporting information, or deposited to a public repository. For example, in addition to summary statistics, the data points behind means, medians and variance measures should be available. If there are restrictions on publicly sharing data—e.g. participant privacy or use of data from a third party—those must be specified.

Reviewer #1: Yes

Reviewer #2: No

4. Is the manuscript presented in an intelligible fashion and written in standard English?

PLOS ONE does not copyedit accepted manuscripts, so the language in submitted articles must be clear, correct, and unambiguous. Any typographical or grammatical errors should be corrected at revision, so please note any specific errors here.

Reviewer #1: Yes

Reviewer #2: Yes

5. Review Comments to the Author

Please use the space provided to explain your answers to the questions above. You may also include additional comments for the author, including concerns about dual publication, research ethics, or publication ethics. (Please upload your review as an attachment if it exceeds 20,000 characters)

Reviewer #1:

 This is an interesting work, and it is well written. I recommend a minor revision for the manuscript.
1. Introduction. This section is well organized, however, the scientific question you are trying to address is not very clear. I feel it is an interesting work, but I do not feel your work is very important. Please highlight your contributions.

**Reply: Thanks. We have stressed our objective of this work by focusing on a county-level city not big cities which have been reported by some literatures. We presented it here and in the revised version.**

**Many studies have focused on the calculation of indicator SDG 11.3.1 in big cities on a regional or global scale. Few studies have focused on county-level cities. The objective of this work is to provide an approach for localizing SDG 11.3.1 to examine land use efficiency at the county level by combining spatial and census data.**

2. Study area. Since you are talking about land use efficiency, please provide more information about the land use in the study area.

**Reply: Thanks. We have added land cover and land use information in the section of the study area as follows and in the revised version.**

**The land cover in Deqing is characterized by 50% mountains, 10% water, and 40% farmland, forming a livable place with mountain and water views[36]. Based on the statistics in 2006, the total land use area in Deqing County was 93,792.82 ha, with agricultural land, including cultivated land, garden land, forest land, and other agricultural land, accounting for 77.59%. Construction land, including urban and rural built-up land, transportation, and water conservancy facilities accounted for 14.30%. Unused land, including lakes, rivers, tidal flats, swamps, and natural reserve regions, accounted for 8.10%.**

3. Results and discussion. You did a good job in describing your research results. However, limited discussion I can see here, this part needs to be improved.

**Reply: Thanks for your suggestions. We have rewritten the section of discussion by pointing out some limitations and the direction of further research based on your suggestions. Please see detail in the revised version and below.**

**A technical flowchart was designed in this study to obtain spatially consistent built-up lands for 1990 to 2015 by integrating four different types of data. By comparing the results with the investigated construction land from the local government, the extracted built-up lands in this work achieved good accuracy. This indicates that high-resolution satellite images, geographical condition data, and land cover products are useful data resources for extracting built-up lands. This work provides an approach to obtain time-series built-up land data with a good accuracy to calculate the land consumption rate and estimate the urban land use efficiency. Because of the high accuracy with the geographical conditions data reflecting the true land cover/land use status, it played a key role in obtaining the built-up lands in different periods. Because of the uncertainty in information extraction from the satellite images, more time was required to integrate the data from different data sources and manually edit the data to ensure that the finally extracted built-up lands for different years can be spatially consistent. As a result, the extracted built-up lands achieved good accuracy, while it was a time-consuming job.**

**The population data we used were the census of the registered household population in a spatial unit of the townships. Due to rapid urbanization, population mobility has become a common phenomenon, so it is difficult to obtain the permanent population of each township that meets the requirements of the time series data. Additionally, according to the monitoring framework (UN habitat, 2018), many villages were not included in the calculation of built-up lands because of the vast distance from the county center, but the registered household population is the entire population in a region of an administrative area, so the population used in this study was not completely consistent with the built-up lands. Therefore, population and the consistency between the population and the geometry of the built-up lands is a key issue in the calculation of SDG 11.3.1, which is also our focus in further studies.**

**The SDG-defined indicator is used for the urban development assessment. The study area is a county center with a much larger rural area than a city. The LCRPGR values for the study site were larger than the average value, indicating that the urban land use efficiency in the country center is lower than the average value of cities worldwide. Compared with small- or medium-sized cities, the study site contains substantial rural areas; it is normal to have relatively less dense construction land areas. This is the primary reason the study site has larger LCRPGR values. From a long-term perspective, it is advantageous that urban land use becomes denser as it grows, which will be beneficial for realizing SDG 11 in the study site.**

**During the calculation of indicator SDG 11.3.1, we determined negative LCRPGR values that were caused by a population decrease during the study period. The negative LCRPGR value indicates that the method given by the UN to calculate the SDG 11.3.1 is specifically for cities suffering from rapid urbanization with positive values for both population growth and land consumption. If there is one negative value in either LCR or PGR, the calculated LCRPGR cannot be included in the computation of the mean LCRPGR value in a region with several cities or a long time span consisting of several short time spans. This study is only concerned with one case; we need more work to test the method of indicator SDG 11.3.1 given by the metadata by investigating more cities in China and worldwide.**

4. Conclusions. The limitation of your work and possible future search directions should also be pointed out here.

**Reply: Thanks. We have rewritten the conclusion section which is also presented below. The possible future research directions are presented in the section of discussion. Please see detail in the revised version and question item 3 above.**

**Satellite imagery, land use, and land cover products can provide continuous and stable data sources to calculate the land consumption rate, population growth rate, and the corresponding land use efficiency in a time series format, although many satellite image processing techniques are required. The designed data processing flowchart can be helpful for others to extract built-up lands.**

**By combining spatial data with statistical data, the SDG11.3.1 indicator can be used to quantitatively analyze the condition of urban land supply and urban land use efficiency as the urban population grows. Generally, the land consumption in Deqing County center has been approximately 3 times faster than the population growth from to 1990–2015, while in the long term, urban land use becomes denser as it grows, which means that land use inefficiency is changing rapidly in the process of urbanization in Wukang, the county center of Deqing. This will have a positive effect on the urban sustainability of the study area.**

Reviewer #2:

 The study adopts the SDG-defined LCGPRG ratio to extract the UN SDG11.3.1 indicator of land use efficiency in a county level in China, and examines its multi-period variations in a 5-year interval. The study design is easy to follow, but the manuscript needs improvement in writing for readers to better understand the context. Major concerns include:
- Introduction: More details about SDG indicators are needed. The SDG indicators are established to assess our environment in global scale. Specifically about urban, Please provide more background on SDG11.

**Reply: Thanks. We have re-organized the part about SDG 11 by adding more information on SDG and SDG 11, and the correspondingly references. Please see detail below and in the revised version.**

**The United Nations Sustainable Development Summit held in September, 2015 adopted a new framework titled "Transforming Our World: The 2030 Agenda for Sustainable Development," which is used to guide the direction of social, economic, and environmental development from 2015 to 2030. The agenda includes 17 sustainable development goals (SDGs), 169 indicators, and 232 indices covering the above three aspects. [11]. These 17 SDGs concern challenges related to poverty, inequality, climate, environmental degradation, prosperity, disaster management, and reducing economic inequality. These factors are interrelated, and the key to achieving one goal often depends on the solution of other goal-related issues. SDG 11, specific for sustainable urban development, is to “make cities and human settlements inclusive, safe, resilient, and sustainable” [12]. Other than the disorderly expansion of cities as mentioned above, rapid urbanization has brought many problems and challenges, including the increasing number of slum dwellers [13,14], increased air pollution [15-17], large volumes of solid waste [18], and insufficient or unaffordable basic services and infrastructure [19], which have made cities more vulnerable to disasters. There are 10 targets and 15 indicators in SDG 11 that address the challenges associated with rapid urbanization and promote the adoption of policies to make cities inclusive, resilient, safe, and sustainable, while these challenges are considerable in many middle-income countries. In this work, we focused on indicator SDG 11.3.1, the ratio (simply LCRPGR) of the land consumption rate (LCR) to population growth (PGR). It was designed to identify land use efficiency [20,21].**

There have been quite a number of global urban settlement databases (both impervious surface and population grid) available online. This manuscript only mentions the GHSL and GPW.

**Reply: We mentioned two of the many available open data source to indicate that these available online data source can be used to calculate the SDG 11 indicators, while this work tries to used statistical population data and our corrected urban built-up land to calculate indicator SDG 11.3.1.**

In Page9 (please note this is the page number of the PDF package, not the manuscript), “…Rozhenkova et al. (2019) found that the current existing databases are insufficient for the purpose of large-scale comparative analysis….” What are these databases? Why are they not sufficient?

**Reply: Thanks. We have clarified this part based on your suggestions as below and in the revised version**

**In terms of a comprehensive scoping review of 23 existing databases, such as the UN Global SDG Indicators Database, World Bank SDG monitoring system, UrbanLex, Open Government Partnership, and ICLEI Local Governments for Sustainability, Rozhenkova et al. (2019) found that the current existing databases are insufficient for the purposes of large-scale comparative analysis because of the significant gap in policy data, especially at the city-level, and the non-system compilation of data covering the SDG 11 target [31]. Therefore, SDG 11.3.1 was categorized as insufficient information available for geospatial analysis (https://unstats.un.org/sdgs/iaeg-sdgs/tier-classification/).**

In Page 10, “…localization is a key problem of SDG indicators…” What does the “localization” mean? Actually, both GHSL and GPW are km-scale global layers. The GHSL is extracted from Landsat imagery per my understanding. What is the rationale that the authors decide to extract their own built-up layers from Landsat imagery instead of using these global products? I believe the 5-year data series is a good justification.

**Reply: The UN SDG goals and the correspondingly indicators were designed originally from global perspective, they may not meet all the requirements of a local SDG study. This causes the problem of localization of SDG indicators. Yes, there are many online available data to support the assessment of SDGs, we need to consider the possible applicability of the available open data sources. In this study, we need to use 6 periods of built-up land data and population data, it is hard to find these data from open data sources, so we need to extract our own built-up data in this work.**

- Study Area: please describe all Data Sets used in the study. It utilizes Landsat imagery, official land use maps from local government, Globelland30 product, and more probably. I guess that the study also utilized the GHSL but could not find any description here. All the information has been scattered in the Methods and Results sections. More details are needed. For example, what is the definition of “artificial surfaces” in Globeland30?

**Reply: Thanks. We discussed the widely used global dataset like GHSL for the calculation of indicator SDG 11.3.1 because of the spatial resolution and the time series problems as what you have mentioned afterward, we did not adopt GHSL in this work. Instead, we proposed a method to extract built-up land for a city with small urban areas like the case in this study.**

**We have added a sub-section of “Data sources” in section of “Materials and methods”. In this new section, we added all of the data we used in this work as table 1, and the descriptions about “artificial surfaces” in Globeland30 and “urban lands” in the multi-temporal global urban land product were presented in the revised version and below.**

**Table 1. Spatial and attribute data we used in this work.**

| **Data/datasets name** | **Data Format** | **Spatial resolution (m)** | **Temporal resolution** |
| --- | --- | --- | --- |
| **County/township boundaries** | **Vector** | **--** | **2015** |
| **Landsat 5 TM** | **Raster** | **30** | **1990,1995,2000,2005,2010** |
| **Landsat 8 OLI** | **Raster** | **30** | **2015** |
| **Globeland30** | **Raster** | **30** | **2000,2010** |
| **Global urban land product** | **Raster** | **30** | **1990,1995,2000,2005,2010** |
| **Geographical condition data** | **Vector** | **--** | **2015** |
| **Township Population** | **Excel** | **--** | **1990,1995,2000,2005,2010,2015** |

**The land cover type of artificial surface in Globeland30 was used in this study. The artificial surface includes various residential areas in cities and townships, industrial and mining lands, transportation facilities, etc. excluding contiguous green spaces and water bodies within the construction land.**

**The term “urban land” used this dataset refers to ‘impervious surface’, i.e., artificial cover and structures such as pavement, concrete, brick, stone and other man-made impenetrable cover types. It has the same meaning as the land cover of artificial surfaces defined in Globeland30.**

It seems that two official land use maps (2010 and 2015) have been utilized in this study (page 14). However, only the 2015 one is mentioned without much details. What does the authors define the built-up land (in other words, how to match the built-up lands in different sources)?

**Reply: We acquired the investigated data of the urban built-up area from the local government with a format of Excel not in maps. We used them mainly for validating the results or the townships areas we extracted. You don’t think it is a true validation so we deleted the comparison and the data. As a supplement, we added a subsection of validation.**

**Because we are focusing on the urban expansion to calculate the urban land consumption rate, so the built-up land in this study is the same as the “artificial surface” defined in Globeland30 and the “urban land” defined in the multi-temporal global urban. We stressed this issue in the section of “Built-up lands extraction” and present here.**

**The urban agglomeration areas need to be acquired before calculating the LCR in Equation (2). Based on the SDG 11 monitoring framework (**[**https://unhabitat.org/ sdg-goal-11-monitoring-framework**](https://unhabitat.org/ sdg-goal-11-monitoring-framework)**), urban agglomeration can be measured as the built-up land. The term “built-up land” has the same meaning as “urban land” in the dataset of Global urban land and “artificial surfaces” in the dataset of Globeland30. Because a 6-year data series of urban lands are needed in this work, no available datasets can meet this requirement. We extracted the built-up lands by integrating the available land cover datasets and manually adjusting them by visually interpreting the satellite images.**

- Methods: I would also suggest that the “Built-up lands extraction” belongs to the Methods section because the authors have made intensive image analysis. Also more details are need in this subsection: what does the “geographical conditions” mean? What are the criteria for manual adjustment (e.g. minimum polygon)? What are the “global urban guilt-up data” in the flowchart in Fig.2? Accuracy assessment is a necessary component but is missing.

**Reply: Thanks. We have moved “Built-up lands extraction” to the section of methods. We have explained “geographical condition data” in the revised version. For making a good accuracy, we adjusted the classified urban lands in open source dataset by overlying the dataset on the satellite imagery and making a correction by deleting wrong classified polygons or adding omission polygons. During the manually correction, we followed the same minimum mapping units of 8*8 pixels which was used in the production of Globeland30.We have replaced “Global urban built-up data” by “Global urban land data” in the revised flowchart. Thanks for pointing this mistake out. Accuracy assessment is added in the revised version as a subsection in the method section.**

### Flowchart to extract the time series built-up lands

**The flowchart for obtaining the built-up lands over the years from 1990 to 2015 with an interval of 5 years is presented in Fig 2.**

**Fig 2. Flowchart for extracting built-up lands in Deqing County.**

**All the satellite images and remotely sensed products were transformed into GCS_China_Geodetic_Coordinate_System_2000 to maintain the same coordinate system. Because of the authority with geographical condition data, which can reflect the distribution of national natural resources and national ecological and environmental conditions in geographic space, built-up land related land use/land cover components in geographic condition data, such as buildings, roads, squares, impervious surfaces, and residential areas, were exported and merged into a single layer, which was regarded as the basis of built-up lands. By aggregating the extracted built-up lands and overlying these on the Landsat OLI false color composition image from 2015, the built-up lands in 2015 were acquired by manually correcting the spatial inconsistency between the built-up lands from the geographical condition data and the actual data from the satellite images, which were identified by visual interpretation. To maintain spatial consistency, the built-up lands from 2015 were used as a reference to obtain the built-up lands for 2010 and 2000. The artificial surfaces from the years 2010 and 2000 were extracted from the Globeland30 dataset. By overlying the Landsat images, the urban land products, and the artificial surfaces from 2010 and 2000, respectively, the built-up lands from 2010 and 2000 were obtained by manually adjusting the classified artificial surfaces to the actual surfaces of the built-up lands reflected from images. With the help of the built-up lands for 2000, 2010, and 2015, the satellite images and urban land products from 2005, 1995, and 1990, we collected the built-up lands for 2005, 1995, and 1990 through manual interpretation and correction. Finally, we obtained built-up lands from 1990 to 2015 with an interval of 5 years to be employed as the urban built-up lands in this study.**

### Validation of the extracted built-up lands

**In order to quantitatively verify the built-up land information extracted in this work, a grid of 3 km × 3 km was created on the study site. Ten percent of the grid was randomly selected, and the built-up regions were manually drawn through a visual interpretation of the satellite images from 1990 to 2015 at 5-year intervals. The interpreted built-up lands in the selected grids for each year were used as the actual ground data. The precision, recall, Intersection over Union (IoU) and accuracies were calculated by constructing an error matrix according to the accuracy assessment method by Olofsson et al. in 2014[44].**

- Methods: “Land consumption Rate” in Page 13, does the land consumption include agricultural and forest lands at all? It will make a big difference of the SGD11.3.1 indicator.

**Reply: we listed the component for the urban land consumption based on the metadata in the method section. Based on the SDG 11.3.1 monitoring framework provided by UN habitat, the urban land area is the urban agglomeration or built-up land area that can be obtained from open data sources.**

- Results: Table 1 in Page 14 is not really accuracy assessment. What are the causal factors of the difference between the investigated and extracted built-up lands? Especially in 2010.

**Reply: Thanks, we have deleted table 1, and added a section about accuracy assessment and the corresponding results in table 2. Please see detail in the revised version and below.**

**To validate the data from this study, we visually interpreted the built-up lands in 10 randomly selected grids of 3 × 3 km for each year as the reference data. The calculated average precision, recall, Intersection over Union (IoU) and accuracies for each year are presented in Table 2. The mean accuracy is higher than 94%, which means that our extracted built-up lands achieved good accuracy.**

**Table 2. Accuracies of our extracted built-up lands in the study site.**

| **Name** | **1990** | **1995** | **2000** | **2005** | **2010** | **2015** |
| --- | --- | --- | --- | --- | --- | --- |
| **Precision** | **85.60** | **62.10** | **71.56** | **67.36** | **73.24** | **80.67** |
| **Recall** | **79.90** | **91.78** | **81.97** | **74.55** | **82.48** | **85.96** |
| **IoU** | **70.43** | **58.83** | **61.83** | **54.77** | **63.38** | **71.27** |
| **Accuracy** | **92.52** | **94.11** | **95.32** | **94.60** | **95.29** | **97.47** |

- “Population Growth” in Page 15-16: Here I have the biggest concern of this study - the validity of PGR. The authors mention that “….There is a decrease in population from 1995 to 2000 (Fig6)…”

**Reply: Thanks for pointing this out. Because of the townships’ merge over years from 1990 to 2015, one of the township was omitted in the calculation of the township population. For making sure the correct of the townships merge and the population, we checked the population number and the township’s relation with the local statistical government. There is an increasing trend of population from 1990-2015, please see the revised Fig 6 below and in the revised version.**

**Fig.6. Change in population in Deqing County from 1990 to 2015**

However, the reasons of this decrease are poorly described in the context. What kind of “local policies”? How do these policies affect the “correspondingly adjustment”? Specifically for Wukang, I could not see the 2000-2005 decreasing trend in Fig.7, which is related to the negative LCRPGR value that is an important piece of conclusion. Actually, Fig.7 shows dramatic 1995-2000 decrease of two townships (Qianyuan and Yuyue) and the similarly dramatic increase in 2000-2005. Note their long-term overall population changes quite low in both townships. What are the causes of these changes? Are these short-term changes meaningful to LCGPRG?

**Reply: Thanks for your questions. We contacted the staff in the statistical department of local government. The reason why there exit a phenomenon of people’s big change from 1995 to 2005 is explained with clear in the revised version and present here.**

**The employed population number is the population registered in the household from the local statistical department. It presented an increasing trend from 1990 to 2015 throughout Deqing County (Fig 6), increasing by 38,000 over the period. Compared with the continuous increase of built-up lands in the county centers and surrounding townships, the demographic changes in townships shifted. From 1990–2000 and 2005–2015, the population in Wukang, the county center, has been increasing (Fig 7). The population changes from 1995 to 2005 were predominantly caused by the adjustment of local administrative divisions. In 1995, the county center was moved from township Qianyuan to Wukang, as a result, many people moved from Qianyuan to Wukang From 1995 to 2000. From 2000 to 2005, a region currently named Fuxi was adjusted to outside the jurisdiction of Wukang, decreasing the population. Generally, people in townships moved to the county center, which caused the rapid urbanization in Wukang.**

**Fig. 7 Variation in population in townships of Deqing from 1990 to 2015**

**As far as the negative LCRPGR value, we think the method is given by UN metadata, we need to point this problem with caution. In the revised version, we discussed this problem and pointing the further research on this issue by large number of cities in our next study.**

Table 2 could serve as a final deliverable of this study. However, why does it only include Wukang? What about other townships? How about the whole county? How about the 1995-2015 long-term land use efficiency?

**Reply: Because the phenomenon of urbanization, most township people have moved to county center to live. Most townships are not real cities not only in population numbers but in size of built-up lands. Many people are living in separated houses in townships, the LCRPGR results can not reflect the land use status of urbanization, so we did not include townships. Wukang is the county center with attractive for people living in the surrounding townships. So we selected Wukang to assess the SDG indicator 11.3.1. Thanks for your suggestions, we have added the long-term land use efficiency from 1990-2005 and 2005-2015 in table 3 of the revised version, and the results showed that the urban land use presents denser as the urban grows, which is positive for the sustainability of the county center.**

- Discussion: It seems to me that the SDG-defined indicator is for urban development assessment. As shown in Fig.1, the study area is actually not urban land. Even for the Wukang township, there is still a much larger rural area than the city. I would suggest the authors discuss the suitability of this SDG11 indicator in rural areas. It could be valuable information for urban SDG community.

**Reply**: **Thanks for your suggestions. We explained the study site is not actually an urban in the section of discussion. Just as what you have said, SDG indicator is primary for urban development assessment. Our study site is a county center facing the phenomenon of urbanization, while other townships are occupied with large areas of rural regions and with many people living in villages. People who are living in the township center are most live in single houses individually. Those townships are far from the definitions on urban by UN. This work is our first try about the SDG indicator 11.3.1. Our next work is on the 500 cities about along the “Silk and Belt”, we will discuss the suitability of the SDG 11 indicator 11.3.1 and the cities sustainability with different sizes. Thanks again for your valuable suggestions.**

6. PLOS authors have the option to publish the peer review history of their article ([what does this mean?](https://journals.plos.org/plosone/s/editorial-and-peer-review-process#loc-peer-review-history)). If published, this will include your full peer review and any attached files.

If you choose “no”, your identity will remain anonymous but your review may still be made public.

Do you want your identity to be public for this peer review? For information about this choice, including consent withdrawal, please see our [Privacy Policy](https://www.plos.org/privacy-policy).

Reviewer #1: No

Reviewer #2: No

[NOTE: If reviewer comments were submitted as an attachment file, they will be attached to this email and accessible via the submission site. Please log into your account, locate the manuscript record, and check for the action link "View Attachments". If this link does not appear, there are no attachment files.]

While revising your submission, please upload your figure files to the Preflight Analysis and Conversion Engine (PACE) digital diagnostic tool, <https://pacev2.apexcovantage.com/>. PACE helps ensure that figures meet PLOS requirements. To use PACE, you must first register as a user. Registration is free. Then, login and navigate to the UPLOAD tab, where you will find detailed instructions on how to use the tool. If you encounter any issues or have any questions when using PACE, please email PLOS at [figures@plos.org](mailto:figures@plos.org). Please note that Supporting Information files do not need this step.
